# Erratum to “Emergency Medical Services resource capacity and competency amid COVID-19 in the United States: preliminary findings from a national survey” [Heliyon 6 (5) (May 2020) Article e03900]

**DOI:** 10.1016/j.heliyon.2020.e05134

**Published:** 2020-10-01

**Authors:** Cody Gibson, Christian Ventura, George Donald Collier

**Affiliations:** a Council on Research and Continuing Education, Health Advocacy and Medical Exploration Society, Inc. Lawrenceville, NJ 08648 USA; b Department of Natural Sciences, Bard College at Simon's Rock, Great Barrington, MA 01230 USA; c Department of Natural Sciences, Calhoun Community College, Tanner, AL 35671 USA

In the original published version of this article, the article listed the order of the authors in the incorrect order: Christian Ventura, Cody Gibson, George Donald Collier. This has now been corrected to: Cody Gibson, Christian Ventura, George Donald Collier. The publisher apologizes for this mistake. Both the HTML and PDF versions of the article have been updated to correct this error.

